# MARK4 Suppression Alleviates Tau Pathology and Neurodegeneration in a Mouse Model

**DOI:** 10.1002/alz70855_099217

**Published:** 2025-12-23

**Authors:** Jun‐qi Huang

**Affiliations:** ^1^ Tokyo Metropolitan University, Hachioji, Tokyo, Japan

## Abstract

**Background:**

Accumulation of abnormally phosphorylated tau proteins is pathological hallmark in a group of neurodegenerative diseases including Alzheimer's disease. In the pathogenesis of these diseases, which are collectively called tauopathies, tau abnormality is thought to cause neurodegeneration. Microtubule affinity‐regulating kinase 4 (MARK4) has been genetically and pathologically associated with Alzheimer's disease and enhance tau phosphorylation and toxicity in Drosophila and mouse traumatic brain‐injury models. We investigated the role of MARK4 on tau pathology and neurodegeneration using a mouse model of tauopathy.

**Method:**

P301S tauopathy model (PS19) mice were crossed with Mark4 knockout mice. Cognitive functions were analyzed via Novel Object Recognition test and the Y‐maze, and tau accumulation and pathology were analyzed by biochemical and histological analyses. To analyze the effects of MARK4 inhibitory peptides, peptides were delivered via Intracerebroventricular injections to mice.

**Result:**

Mark4 deletion improved survival rates and cognitive functions of PS19 mice. These phenotypes were accompanied by reduced neurodegeneration and astrogliosis. Mark4 deletion lowered accumulation of pathological forms of tau, such as those phosphorylated at Ser356, AT8‐positive tau and thioflavin S‐positive tau. These results suggesting that MARK4 inhibition may be a therapy for tauopathies.

We also created a highly selective and cell‐permeable peptide inhibitor. This peptide inhibited MARK4, but not the closely related kinase, MARK2. ICV injection of the peptides at the concentration of 0.1mg/ml caused no severe abnormalities in wild‐type mice.

**Conclusion:**

MARK4 is critically involved in tau‐mediated neurodegeneration. Further characterization of the effects of MARK4‐specific inhibitors may lead to a disease‐modifying strategy for tauopathies.

